# Deoxyamphimedine, a Pyridoacridine Alkaloid, Damages DNA via the Production of Reactive Oxygen Species

**DOI:** 10.3390/md7020196

**Published:** 2009-05-25

**Authors:** Kathryn M. Marshall, Cynthia D. Andjelic, Deniz Tasdemir, Gisela P. Concepción, Chris M. Ireland, Louis R. Barrows

**Affiliations:** 1 Department of Pharmacology and Toxicology, University of Utah, 30 South 2000 East Rm. 201, Salt Lake City, Utah 84112, USA; E-Mail: andjelic4@hotmail.com (C-D.A.); 3 Department of Medicinal Chemistry, University of Utah, 30 South 2000 East Rm. 301, Salt Lake City, Utah 84112, USA; E-Mail: cireland@pharm.utah.edu (C-M.I.); 5 The Marine Science Institute, University of Philippines, Velasquez Street, Dilman, Quezon City 1101, Philippines; E-Mails: giselle@upmsi.ph (G-P.C.)

**Keywords:** deoxyamphimedine, neoamphimedine, amphimedine, pyridoacridine, reactive oxygen species

## Abstract

Marine pyridoacridines are a class of aromatic chemicals that share an 11*H*-pyrido[4,3,2-*mn*]acridine skeleton. Pyridoacridine alkaloids display diverse biological activities including cytotoxicity, fungicidal and bactericidal properties, production of reactive oxygen species (ROS) and topoisomerase inhibition. These activities are often dependent on slight modifications to the pyridoacridine skeleton. Here we demonstrate that while structurally similar to neoamphimedine and amphimedine, the biological activity of deoxyamphimedine differs greatly. Deoxyamphimedine damages DNA *in vitro* independent of topoisomerase enzymes through the generation of reactive oxygen species. Its activity was decreased in low oxygen, with the removal of a reducing agent and in the presence of anti-oxidants. Deoxyamphimedine also showed enhanced toxicity in cells sensitive to single or double strand DNA breaks, consistent with the *in vitro* activity.

## 1. Introduction

Marine pyridoacridines are a class of planar aromatic molecules with an 11*H-*pyrido-[4,3,2*mn*]acridine skeleton that show affinity for DNA. Great interest in this class of compounds is evident from numerous synthetic studies and total synthesis reports [1–8]. In general, pyridoacridines are cytotoxic and some of them possess potent anti-viral, anti-fungal, anti-bacterial, anti-tumor and anti-parasitic activity [9]. For the majority of this class, cytotoxicity has been shown to be due to DNA-binding properties, topoisomerase inhibition and the production of reactive oxygen species (ROS) [9].

**Figure 1:**
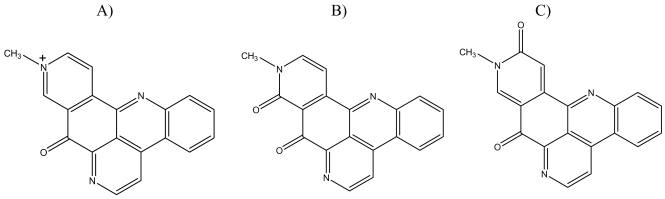
Chemical structures of: A) deoxyamphimedine, B) neoamphimedine and C) amphimedine.

Deoxyamphimedine, neoamphimedine and amphimedine are pyridoacridines isolated from *Xestospongia* sponges collected in the Philippines and Micronesia (Figure 1). We previously reported that neoamphimedine is cytotoxic via topoisomerase 2-dependent DNA aggregation/catenation, while amphimedine is relatively non-toxic [10]. During the analysis of the bioactivities of neoamphimedine and amphimedine it was observed that deoxyamphimedine cleaved DNA *in vitro* in the absence of topoisomerase (1 or 2) [11]. In this paper we describe the ability of deoxyamphimedine to damage DNA through the production of ROS.

Reactive oxygen species, although produced during normal aerobic metabolism in mammalian cells, have been implicated in cell and tissue damage [12,13]. Free radicals produced by oxidative processes can attack DNA at bases or sugars, causing primarily single strand breaks, as well as, secondary double strand breaks and abasic sites [14–16]. Quinone functions are capable of redox cycling. One electron reduction of the quinone forms reactive semiquinones, allowing electron transfer to other species [17]. Higher redox potential of quinone molecules has been correlated with increased DNA cleavage [18]. This study demonstrates the cytotoxicity of deoxyamphimedine, its ability to intercalate and damage DNA and the attenuation of this activity when the generation of ROS was inhibited.

## 2. Results and Discussion

### 2.1. Cytotoxicity in Cultured Mammalian Cell Lines

Pyridoacridines are often cytotoxic in cultured cell lines. Deoxyamphimedine was originally found to be toxic in HCT-116 (human colon tumor) cells and shown to have enhanced toxicity (4-fold) against the EM9 strain of Chinese hamster ovary (CHO) cells, which are deficient in single strand (ss) DNA break repair when compared to the wild type AA8 strain [11]. The sensitivity observed in ssDNA break repair deficient cells typically correlates with compounds having topoisomerase 1 or reactive oxygen species (ROS) mediated DNA cleavage [19–21]. We further tested deoxyamphimedine in a panel of other cultured cell lines previously tested with neoamphimedine and amphimedine [10]. The IC_50_ values of deoxyamphimedine are reported in Table 1.

The observed IC_50_ values were typically slightly lower than those reported for neoamphimedine, while amphimedine was not toxic up to 100 μM tested [10]. Deoxyamphimedine and neoamphimedine were cytotoxic in every tumor cell line examined, indicating their potential as anti-cancer drugs. Deoxyamphimedine was almost equally toxic to the A2780wt and its paired multi-drug resistant cell line, A2780AD, which exhibited resistance to doxorubicin (33 fold), M-AMSA (8 fold), and taxol (15 fold) when compared to the A2780wt cell line. The lack of significant fold difference for deoxyamphimedine in the A2780 cell lines indicates that it is likely not a substrate for the multi-drug resistant pump that often impedes the efficacy of cancer drugs in chemotherapy. In repeated experiments, deoxyamphimedine again was selectively more toxic to the mutant CHO cell lines, EM9 and xrs-6, than the wild type (AA8). The EM9 cell line has enhanced sensitivity to topoisomerase 1 poisons and ROS that cause DNA single-strand breaks. Sensitivity observed in the xrs-6 cell line is often associated with compounds that generate DNA double strand breaks via topoisomerase 2 or high amounts of single strand DNA cleavage [22]. These data confirm DNA strand breakage as a contributing mechanism of deoxyamphimedine cytotoxicity.

### 2.2. Deoxyamphimedine Intercalates DNA

**Figure 2:**
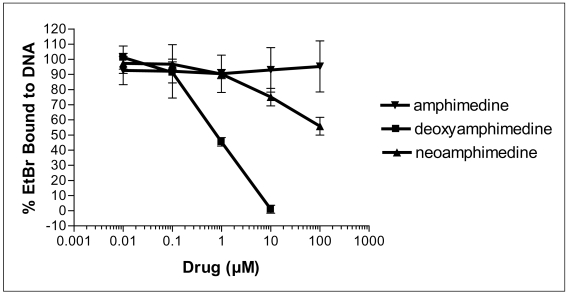
EtBr displacement by the amphimedines as a measure of DNA intercalation. Values are the average of n=4 ± SD.

Many pyridoacridines have shown binding affinity for DNA, and this binding often correlates with the observed cytotoxicity [9]. To measure DNA intercalation, deoxyamphimedine was compared to amphimedine and neoamphimedine, in an ethidium bromide (EtBr) displacement assay. Deoxyamphimedine exhibited potent DNA intercalation, displacing 50% of EtBr at 1 μM, while neoamphimedine required approximately 100 μM to displace 50% of EtBr. Amphimedine was unable to significantly displace EtBr at concentrations up to 100 μM (Figure 2). These results suggest that deoxyamphimedine has a higher affinity for DNA than its analogs, as might be expected from its positive charge.

### 2.3. Deoxyamphimedine Cleaves DNA Independently of Topoisomerase 1 or 2

DNA intercalators often interfere with DNA metabolizing enzymes, such as the topoisomerases. Therefore, *in vitro* analyses to evaluate the potential importance of the topoisomerase enzymes for DNA damage were performed.

**Figure 3:**
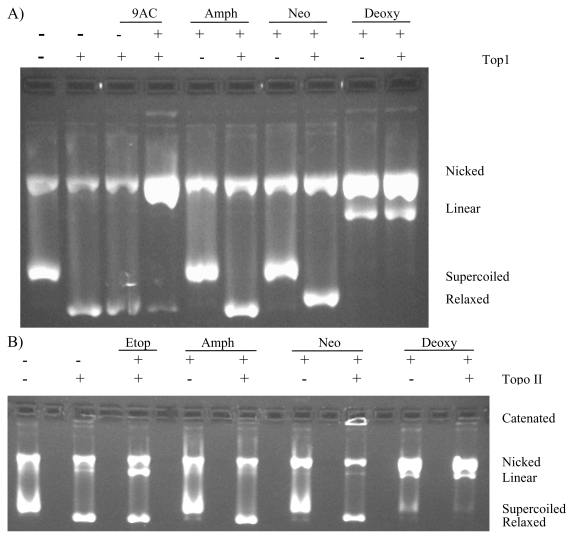
Topoisomerase 1 and topoisomerase 2 DNA cleavage gels for amphimedine (Amph), neoamphimedine (Neo) and deoxyamphimedine (Deoxy). A) Plasmid DNA (supercoiled rf M13 mp 19 DNA) was used in the cleavage assay in the presence or absence of topoisomerase 1, with DMSO, 9-aminocamptothecin, 9AC (9 μM) that induces topoisomerase 1-mediated DNA single strand breaks, amphimedine (100 μM), neoamphimedine (100 μM), and deoxyamphimedine (100 μM). B) Plasmid DNA was used in the cleavage assay in the presence and absence of topoisomerase 2, etoposide (Etop, 100 μM) that induces topoisomerase 2-mediated DNA double strand breaks, amphimedine (100 μM), neoamphimedine (100 μM), and deoxyamphimedine (100 μM).

The topoisomerase 1 and 2 DNA cleavage assays (Figure 3) showed that amphimedine did not inhibit either of the topoisomerase enzymes, nor did it increase the amount of cleaved DNA observed in the gel. Neoamphimedine, as previously reported, has no effect on topoisomerase 1, but produced catenated DNA complexes in the presence of topoisomerase 2, inducing only minimal topoisomerase 2-mediated DNA cleavage [10]. Deoxyamphimedine was a potent cleaver of DNA *in vitro* as indicated by the increased intensity in the nicked (single strand break) and linear (double strand break) plasmid DNA bands. This activity was independent of the presence of topoisomerase 1 or 2.

**Figure 4:**
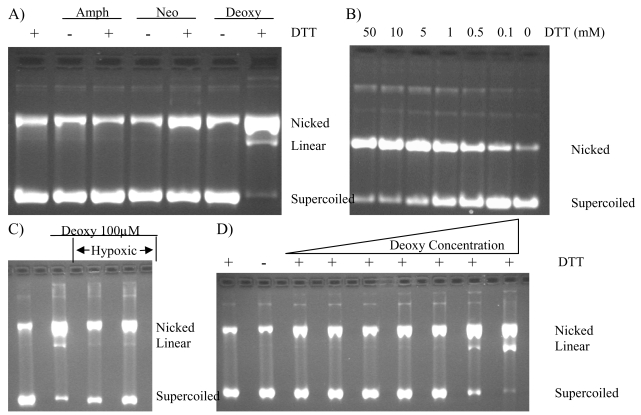
Deoxyamphimedine dependence on DTT, oxygen and concentration. A) Plasmid DNA was incubated in the presence of DTT (5 mM), amphimedine (Amph, 100 μM), neoamphimedine (Neo, 100 μM), and deoxyamphimedine (Deoxy, 100 μM). B) Plasmid DNA was incubated in the presence of deoxyamphimedine (100 μM) with varying amounts of DTT (as shown). C) Plasmid DNA was incubated in the presence of DTT (5 mM) and deoxyamphimedine (100 μM) as indicated. Hypoxic conditions were induced in the last 2 lanes by bubbling argon in the reaction vessel. D) Plasmid DNA was incubated in the presence or absence of DTT (5 mM) with varying amounts of deoxyamphimedine (0.1 μM, 0.5 μM, 1 μM, 5 μM, 10 μM, 50 μM, 100 μM). Increase in cleaved plasmid DNA over control in this experiment was quantifiable at 5 μM (12%), 10 μM (14%), 50 μM (67%), and 100 μM (84%).

Further evaluation in the cleavage assay showed deoxyamphimedine could fully cleave DNA under aerobic conditions in experiments carried out in water to which only dithiothreitol (DTT, a reducing agent) and DNA were added. Figure 4A shows results for all of the amphimedines in the absence or presence of 5 mM DTT. While no catenation was observed with neoamphimedine, there was a slight increase in the nicked DNA band. The amount of cleaved DNA observed was greatly increased with deoxyamphimedine in the presence of DTT. The amount of deoxyamphimedine-induced DNA cleavage observed increased with increasing amounts of DTT (Figure 4B). However, under hypoxic conditions the amount of induced DNA cleavage was attenuated (Figure 4C). The extent of DNA cleavage increased with increasing deoxyamphimedine concentrations (Figure 4D) with detectable cleavage appearing at 5 μM and increasing to approximately 84% cleavage at 100 μM in this experimental system. Additional studies using the cleavage assay showed that deoxyamphimedine-induced DNA cleavage exhibited a time dependency (data not shown), with complete cleavage of the substrate in the presence of 5 mM DTT within 30 minutes. Deoxyamphimedine also cleaved plasmid DNA, regardless of its topological state. Specific reaction conditions, such as high salt or temperature, will reverse DNA damage detectable from stabilized drug-top1-DNA complexes, but not ROS-induced DNA damage. These conditions did not reverse deoxyamphimedine-induced DNA damage (data not shown).

### 2.4. Protection of DNA from Deoxyamphimedine Cleavage

**Figure 5:**
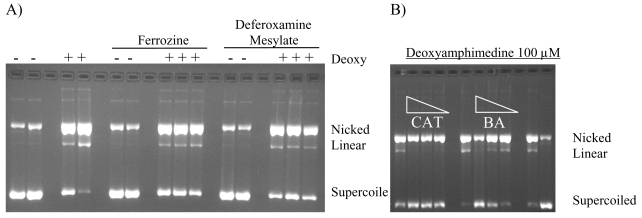
A) Effects of chelators on deoxyamphimedine (Deoxy)-induced DNA cleavage. Plasmid DNA was incubated in the absence or presence of deoxyamphimedine (100 μM), ferrozine (100 μM) and deferoxamine mesylate (100 μM). B) Anti-oxidant enzymes and radical scavenger protection of deoxyamphimedine induced DNA cleavage. Plasmid DNA was incubated in the presence of deoxyamphimedine (100 μM) and varying amounts of catalase (CAT) (88 u, 8.8 u, 0.88 u) or the free radical scavenger benzoic acid (BA) (8.8 mM, 0.88 mM, 0.088 mM).

Metal chelators and FeSO_4_ were used to determine if a Fenton type reaction could be responsible for deoxyamphimedine-induced DNA cleavage. Metal chelators were added to aerobic reactions containing deoxyamphimedine, DTT and DNA. DNA cleavage in the presence of deoxyamphimedine was not further stimulated when FeSO_4_ (100 μM) was added (data not shown). Ion coupled plasma-mass spectrometry (ICP-MS) was performed on deoxyamphimedine in DMSO to determine if it bound to metal ions in solution. The results suggest that some metal binding may be occurring (data not shown). However, only slight protection from DNA cleavage was sporadically detectable in the presence of deferoxamine mesylate (up to 50 mM) and ferrozine (up to 100 μM) (Figure 5A) or EDTA (100 μM) (data not shown). Overall, the addition of metals or metal chelators did not strongly alter the DNA cleaving ability of deoxyamphimedine.

To determine if reactive oxygen species played a role in deoxyamphimedine’s DNA cleavage, an anti-oxidant enzyme and radical scavengers were employed to attenuate DNA cleavage. Figure 5B shows that catalase (CAT) and benzoic acid (BA) were able to inhibit cleavage by deoxyamphimedine. Other radical scavengers, glutathione (GSH) and N-acetylcholine (NAC), also attenuated deoxyamphimedine-induced DNA cleavage (data not shown). Superoxide dismutase (SOD), which converts superoxide to H_2_O_2_, did not decrease deoxyamphimedine-induced DNA cleavage. No additional protection was detected when SOD was used with catalase when compared to catalase alone (data not shown).

### 2.5. Discussion

Although amphimedine, deoxyamphimedine and neoamphimedine are chemically very similar (see Figure 1), our data shows that their minor structural differences significantly alter their biological activities. Amphimedine was relatively inactive compared to deoxyamphimedine and neoamphimedine, both of which demonstrated considerable DNA-directed activity. Amphimedine did not significantly intercalate DNA at the concentrations tested and was not cytotoxic at doses up to 100 μM. Neoamphimedine and deoxyamphimedine displace EtBr from DNA, although deoxyamphimedine is more effective than neoamphimedine. Both deoxyamphimedine and neoamphimedine are cytotoxic, although by differing mechanisms. Deoxyamphimedine is a positively charged compound, which differentiates it from amphimedine and neoamphimedine. As mentioned above, it interacts with DNA avidly and is the most cytotoxic of the three amphimedines tested. Several other marine pyridoacridines (e.g., dercitin, cystodytin J, ascididemin and diplamine) have previously been shown to intercalate DNA [23–25].

Deoxyamphimedine facilitated DNA cleavage *in vitro*, unlike amphimedine or neoamphimedine. Under aerobic conditions, the reducing agent DTT was the sole requirement for full activity. Low oxygen conditions reduced the amount of DNA cleaved and the total absence of DTT yielded undetectable levels of DNA damage. The DNA cleavage was time dependent, requiring more time to reach maximal DNA damage than is typically needed by topoisomerase 1-DNA cleavable complex stabilizing drugs (DNA, topoisomerases and topoisomerase poisons form the ternary complex very rapidly).

In our experimental systems, deoxyamphimedine produced results very similar to ascididemin, another pyridoacridine that produces damage to DNA through ROS [24,26]. Anti-oxidants and anti-oxidant enzymes protected DNA from deoxyamphimedine-induced cleavage, indicating a ROS-mediated mechanism. Extensive protection against DNA cleavage by the anti-oxidant enzyme catalase, suggested H_2_O_2_ as a likely intermediate ROS. In addition, anti-oxidants and reactive scavengers including glutathione, BHA, NAC and benzoic acid all protected against DNA damage. Therefore, the production of ^−^OH most likely occurs. Previous work using electron paramagnetic resonance spectroscopy with ascididemin and analogs yielded a complex signal that indicated multiple iminoquinone radical species were likely formed [24]. We speculate that this would be the case for deoxyamphimedine as well.

Quinones and iminoquinones are capable of redox cycling. One electron reduction of the quinone forms a reactive semiquinone, allowing electron transfer to other species [27]. Higher redox potential of quinone molecules has been correlated with increased DNA cleavage [18,28]. Moreover, evidence suggests that doxorubicin and daunorubicin, both with quinone moieties, owe some of their clinical activity to ROS generation [27,29–33]. We hypothesize that it is the iminoquinone portion of deoxyamphimedine that is responsible for the redox cycling and ROS generation. Redox cycling and ROS generation require a source of oxygen. Under hypoxic conditions the ability of deoxyamphimedine to cleave DNA diminishes. Direct reduction of deoxyamphimedine to the semi-iminoquinone species likely facilitates the production of H_2_O_2_ and DNA damaging free radicals. It will be beneficial to further characterize the redox potential of deoxyamphimedine when more of it is available, current supplies being exhausted. All together, these data strongly implicate ROS as the mediator of deoxyamphimedine-induced DNA damage.

## 3. Experimental

### 3.1. Chemicals and Reagents

Isolation and chemical characterization of amphimedine, deoxyamphimedine and neoamphimedine have been described [11,34]; all were greater than 95% pure. They were isolated from two *Xestospongia* sp. sponges collected from Surigao, Philippines and Palau. All chemicals and reagents were obtained from Sigma Chemical Co. (St. Louis, MO, USA) or Baker Chemical Co. (Springfield, NJ, USA) unless otherwise noted. Drug standards were purchased from Sigma Chemical Co. Radioactive thymidine was purchased from New England Nuclear (Boston, MA, USA). Restriction enzymes and buffers were purchased from New England Biolabs (Ipswich, MA, USA). Radiolabeled (4.4 × 10^3^ cpm/g) ^3^H replicative form (rf) of M13 mp19 was isolated by the alkaline lysis method as described [35,36]. Human topoisomerase 1 and topoisomerase 2 were isolated as described [36,37].

### 3.2. Assessment of DNA Intercalation

DNA intercalation was measured as the ability of drug to displace ethidium bromide (EtBr) from DNA. Based on the protocol of McDonald *et al.* [25], 10 serial dilutions (nM-μM) of drug were added with 0.5 μM EtBr to 0.5 μM salmon testes DNA (Sigma Chemical Co.) in a 96-well microtiter plate and incubated for 30 min at room temperature. A fluorimeter was used to quantify the fluorescence of each reaction, (280 nm excitation wavelength; displacement of EtBr was measured as a decreased fluorescence at 600 nm).

### 3.3. Quantitation of DNA and Cleavage

DNA cleavage assays were performed as previously described [10,36]. In brief, 20 μL volumes containing 50 mM Tris-HCl (pH 7.5), 85 mM KCl, 10 mM MgCl_2_, 0.5 mM EDTA, 30 μg/mL bovine serum albumin (Atlanta Biologicals, Atlanta, GA), 2 mM DTT, 500 ng of radiolabeled and supercoiled rf M13 mp 19 DNA and 100–150 *ng* purified topoisomerase 1 or 80–120 ng purified topoisomerase 2 were treated with 1–2 μL drug (in DMSO) and incubated at 30°C for 30 min. The reactions were stopped by the addition of 2 μL of 1.5 mg/mL proteinase K in 0.5% SDS and incubated at 37°C for 60 min. The DNA was fractionated by electrophoresis in 0.8% agarose (containing 50 ng EtBr/mL TAE) to separate the nicked, linear, relaxed and supercoiled isomers.

EtBr stained DNA was visualized by its fluorescence under UV light, and the bands were sliced out of the gel. To quantify the DNA in the bands the gel slices were placed in scintillation vials with 1 mL water, melted in a microwave oven and mixed with 10 mL Opti-Fluor (Packer Co., Meridien, CT, USA) while molten. Radioactivity determined by *standard* liquid scintillation counting [37]. The percentage of cleaved DNA, represented by radioactivity in the combined nicked and linear bands, was determined relative to the total radioactivity in the reactions, following subtraction of radioactivity due to endogenously cleaved DNA. The percentage of catenated DNA was determined similarly. All experiments were repeated three times, representative results are presented.

### 3.4. Cell Culture

The human epidermoid-nasopharyngeal tumor cell line (KB), human melanoma cell line (SK-mel-5), human breast cancer cell line (MCF7), human ovarian multi-drug resistant cell lines (A2780 and A2780AD), and Chinese hamster ovary cell lines (CHO): AA8 (wild type), EM9 (single strand break repair deficient) and xrs-6 (double strand break repair deficient) were used. KB, SK-mel-5, MCF7, EM9 and AA8 cell lines were purchased from ATCC, Rockville, MD). A2780 and A2780AD cell lines were provided by Dr. Jindrich Kopecek (University of Utah, UT, USA) and the xrs-6 cell line was a generous gift from Dr. Penny A. Jeggo and co-workers (University of Sussex, U.K.). All cell lines were maintained in Minimal Essential Medium alpha modification (α-MEM), Atlanta Biologicals. CHO cell lines were supplemented with 10% fetal bovine serum (FBS) (Atlanta Biologicals), and the others were supplemented with 2.5% FBS and 7.5% calf sera (Atlanta Biologicals), 100 u/mL penicillin and 100 μg/mL streptomycin (Sigma Chemical Co.). Cells were grown at 37°C as monolayers in 75 cm^2^ culture flasks and detached with trypsin before seeding into microtiter culture plates.

### 3.5. MTT Cell Cytotoxicity Assay

Cytotoxicity was established in a 3-(4,5-dimethylthiazol-2-yl)-2,5-diphenyltetrazolium bromide (MTT) assay as performed by Mossman [38] and modified by others [22, 39, 40]. Drugs were dissolved in 100% DMSO at initial concentrations of 10 mM and serially diluted. The final concentration of DMSO in the cell culture wells was 1% or less. Cells were seeded in 200 μL of growth media in Corning 96-well microtiter plates at 30,000 cells/well (AA8), 40,000 cells/well (xrs-6 or EM9), or 20,000 cells/well (human cell lines). Four hours after seeding, cells were treated, each dose in quadruplicate, with 2 μL of drug (CHO cell lines) or 1 μL of drug (all other cell lines). CHO cell lines were refed at 18 hours. After 72 hours, all cultures were refed with 100 μL McCoy’s medium and 11 μL MTT (5 mg/mL in PBS, pH 7.4) was added to each well. The plates were incubated for 4 hours at 37°C. Viable cells reduced MTT to a purple formazan product that was solubilized by the addition of 100 μL DMSO to aspirated culture wells. The absorbance at 540 nm was measured for each well using a Bio-Rad MP450 plate reader (Bio-Rad, Hercules, CA). Following subtraction of absorbance from blank culture cells, the average absorbance for each set of drug-treated wells was compared to the average absorbance of the control wells to determine the fractional survival at any particular drug concentration. The inhibitory concentration 50 (IC_50_) was defined as the drug concentration that yielded a fractional survival of 50%.

### 3.6. Time and Concentration Dependence of DNA Cleavage

To determine the time and concentration dependency of deoxyamphimedine cleavage *in vitro*, DNA cleavage assays were performed as described above (*3.3*) using different time points of incubation (0 to 60 minutes) and increasing concentrations of deoxyamphimedine (0.1 to 500 μM).

### 3.7. DNA Cleavage Protection Assays

Protection from deoxyamphimedine-induced DNA cleavage was determined as described [24]. Metal chelators (i.e. EDTA, ferrozine, deferoxamine mesylate), the metal salt, FeSO_4_, radical scavengers [i.e. glutathione (GSH), *N*-acetylcysteine (NAC) and benzoic acid (BA)] or anti-oxidant enzymes [i.e. catalase (CAT)] were added to reactions described above to further define the potential mechanisms of DNA cleavage.

### 3.8. Assessment of DNA Cleavage under Hypoxic Conditions

DNA cleavage reactions were carried out as described above (*3.3*); however, these reactions were modified to be hypoxic based on the work of Radisky *et al*. [41]. Drug and reaction mix containing DNA were bubbled with argon for several minutes prior to the addition of drug to the reaction mix. At this point, the reaction mix was bubbled under argon for 1 min, lids were closed and it was then incubated at 37 °C for 30 min before being run out on an agarose gel, as described above.

## Figures and Tables

**Table 1:** Cytotoxicity of Deoxyamphimedine.

Cultured Mammalian Cell Line	IC_50_ in μM
Human melanoma, SK-mel-5	5.5
Human epidermoid-nasopharyngeal cancer, KB	3.8
Human breast cancer, MCF7	5.9
Human ovarian cancer-wild type, A2780wt	0.3
Human ovarian cancer-multi-drug resistant, A2780AD	0.4
Chinese hamster ovary, CHO wild type, AA8	13.7
CHO-double strand (ds) DNA break repair deficient, xrs-6	4.2
CHO-single strand (ss) DNA break repair deficient, EM9	3.8
